# MiR-107 function as a tumor suppressor gene in colorectal cancer by targeting transferrin receptor 1

**DOI:** 10.1186/s11658-019-0155-z

**Published:** 2019-05-16

**Authors:** Yuxiang Fu, Liewen Lin, Ligang Xia

**Affiliations:** Department of Gastrointestinal Surgery, Shenzhen People’s Hospital, The Second Clinical Medical College of Jinan University, The First Affiliated Hospital of South University of Science and Technology, Shenzhen, 518055 China

**Keywords:** Colorectal cancer, microRNA107 (miR-107), Cancer progress, Transferrin receptor 1 (TFR1)

## Abstract

**Background:**

While microRNAs (miRNAs) are known to play a critical role in the progression of colorectal cancer, the role of miR-107 remains unknown. We evaluated its role and explored the underlying mechanism.

**Materials & methods:**

MTT, wound-healing, transwell migration and transwell invasion assays were performed to evaluate the role of miR-107 in SW629 cell proliferation, migration and invasion. Real time-PCR and dual-luciferase reporter gene, TFR1 overexpression and western blotting assays were used to explore the underlying mechanism.

**Results:**

MiR-107 is downregulated in colorectal cancer tissues and several human colorectal cancer cell lines. Low miR-107 expression often indicates a poor survival rate for colorectal cancer patients. MiR-107 suppresses the proliferation, migration and invasion of SW620 cells by negatively regulating transferrin receptor 1 (TFR1).

**Conclusion:**

MiR-107 suppresses the metastasis of colorectal cancer and could be a potential therapy target in colorectal cancer patients.

**Electronic supplementary material:**

The online version of this article (10.1186/s11658-019-0155-z) contains supplementary material, which is available to authorized users.

## Introduction

Colorectal cancer, also known as colon cancer, is one of the most common human malignancies and one of the leading causes of cancer-related mortality worldwide [1, 2]. Chemotherapy combined with surgery remains the main therapy strategy for colorectal cancer. Although many advances have been made in developing novel therapeutics, it remains an incurable disease, mainly due to its multidrug resistance (MDR) to chemotherapy agents [3, 4]. It is reported that about 50% of patients that undergo surgical resection and aggressive chemotherapy have a recurrence of the disease [5]. Tumor progression and cell metastasis are multi-step processes that involve several mechanisms. They are regarded as the main factors that result in MDR [6, 7]. Further understanding of the molecular mechanisms in colorectal cancer is necessary for developing new therapeutic approaches to improve patient prognosis.

MicroRNAs (miRNAs) are a class of non-coding RNAs that play central roles in many processes in cell biology, including proliferation, migration, invasion and differentiation [8, 9]. They typically regulate cellular processes by directly interacting with the 3′-untranslated region (3′-UTR) of corresponding target messenger RNA (mRNA), leading to translational inhibition or degradation [10]. MiRNAs are involved in many tumor processes, such as development, metastasis, drug resistance and recurrence. They are excellent prospects as biomarkers for cancer diagnosis and therapy [11, 12]. For example, miR-34, one of most studied miRNAs, plays an important role in tumor development and cancer progress and is now an attractive target for tumor therapy. MRX34, a liposomal formulation of miR-34a, has been targeted in preclinical and clinical cancer therapy experiments [13, 14].

Considerable evidence shows that numerous miRNAs are involved in the progression of colorectal cancer. MiRNA-210, miRNA-21 and miRNA-126 have identified as diagnostic biomarkers for colorectal cancer [15]. MiRNA-21 has been shown to modulate cell cycle progression in colorectal cancer cells. MiR-15/16, miR-133, miR-143, miR-1 and miR-370 also contribute to colorectal cancer processes [15–18]. Recently, miR-107 has been identified as a key factors for cell proliferation and angiogenesis in colorectal cancer, but the underlying mechanism is still largely unknown [19, 20].

In this study, we found that miR-107 was downregulated in human colorectal cancer tissues and human colorectal cancer cell lines and that its expression is negatively correlated with the survival rate of colorectal cancer patients. Further studies showed that miR-107 suppressed the proliferation, migration and invasion of SW620 cells by targeting transferrin receptor 1 (TFR1). Our study revealed that miR-107 functions as a tumor suppressor in colorectal cancer and that targeting miR-107 may inhibit colorectal cancer metastasis.

## Materials and methods

### Materials

Dulbecco’s modified Eagle medium (DMEM), RPMI-1640 medium, fetal bovine serum (FBS), penicillin–streptomycin (PS) and Lipofectamine LTX & PLUS reagents were purchased from Thermo Fisher Scientific. Transwell plates (6.5 mm) with an 8.0-μm pore polycarbonate membrane insert were obtained from Corning. Matrigel was purchased from BD Biosciences. The Firefly & Renilla Dual Luciferase Assay Kit was from Sino Biological Inc. The E.Z.N.A. Total RNA Kit I was from Omega Bio-Tek. The RT-PCR kit was from Life Technologies. NC mimic and MiR-107 mimic were synthesized by RiboBio. Antibodies against TFR1 and β-tubulin were obtained from Cell Signaling Technology. Other reagents were from Sigma-Aldrich.

### Cell culture

The human colorectal cancer cell lines LOVE, SW620, SW480, HT29 and DLD-1 were purchased from the American Type Culture Collection (ATCC). Human normal colon epithelial cell HCoEpiC was also obtained from the ATCC and cultured in RIPM-1640 supplemented with 10% FBS, 100-U/ml PS, and 100 mg/ml streptomycin sulfate. All the colorectal cancer cells were maintained in DMEM supplemented with 10% FBS, 100-U/ml PS and 100 mg/ml streptomycin sulfate. All the cells were cultured at 37 °C in a humidified air atmosphere containing 5% CO_2_.

### Patients and tumor tissues

We collected 50 paired samples of human colorectal cancer tissue and corresponding normal mucosa tissue from patients undergoing surgery at Shenzhen People’s hospital, Shenzhen, China. The tissues were snap-frozen in liquid nitrogen. The study was approved by the Ethics Committee of Shenzhen People’s Hospital and supervised by the hospital. All the patients gave informed consent for the use of their specimens before sampling.

### RNA extraction and RT-PCR

We analyzed the miR-107 and TFR1 mRNA of cells and tissues using RT-PCR as described previously [21]. Briefly, total RNA was extracted from the cells and tissues with Trizol reagent following the manufacturer’s instructions (Invitrogen). Then a Transcriptor First Strand cDNA Synthesis Kit was used for reverse transcription of total RNA to obtain cDNA. After that, real-time PCR was then performed using the iQ SYBR Green Supermix Kit (Bio-Rad). Relative gene expression was calculated using the comparative cycle threshold (Ct) method [22]. Each sample was run in triplicate and glyceraldehyde 3-phosphate dehydrogenase (GAPDH) was used as an internal control. The oligonucleotide primers were: miR-107 forward 5′-TGTGTAGTAGTTTGTTTATAGTG′ and reverse 5′-CCAACTCTACAACTACTAAATC-3′; TRF1 forward 5′-GCAGGATGAAGGGAGGACAC-3′ and reverse 5′-GCGATCTGTCAGAGCACCTC-3′; and GADPH forward 5′-GTGAACCATGAGAAGTATG-3′ and reverse 5′-CGGCCATCACGCCAC AGTTTC-3′. The primers, SYBR Green I Master Mix and DNA templates were mixed to form a PCR system. The PCR conditions were: 45 cycles of 95 °C for 10 s, 60 °C for 20 s and 72 °C for 20 s. PCR was performed with the ABI PRISM 7900 Sequence Detection System (Applied Biosystems). The experiment was conducted independently in three biological replicates.

### Cell proliferation assay

The 3-(4, 5-dimethylthiazol-2-yl)-2, 5-diphenyltetrazolium bromide (MTT) assay was used to determine cell viability. Cells (1 × 10^6^ for cell viabilities after 24 h; 0.7 × 10^6^ for 48 h; 0.5 × 10^6^ for 72 h; and 0.3 × 10^6^ for 96 h) suspended in 100 μl cell growth medium were seeded at 96-well plates, cultured for 24 h, then treated with NC mimic or miR-107 mimic for 24, 48, 72 or 96 h. After the desired time point, the cells were incubated with 4% MTT for another 4 h, and absorbance was detected at 490 nm on a Multi-Detection Microplate Reader (BMG Labtech).

### Wound healing assay

5 × 10^5^ cells suspended in 0.6 ml cell growth medium were seeded in 6-well plates and cultured for 24 h until approximately 100% confluence. The cells were scratched with a 10-μl pipette tip and then washed three times with PBS. After that, the cells were treated with NC mimic or miR-107 mimic for another 8 h in 2% serum medium. Images of the same field were taken at 0 and 8 h (0 h is when the NC mimic or miR-107 mimic were added) with an Olympus IX70 inverted microscope. Migratory cells were quantified using Image-Pro Plus 6.0 software. Three independent experiments were conducted.

### Transwell migration assay and transwell invasion assay

The transwell migration assay and transwell invasion assay were conducted with a Corning Inc. transwell chamber. In the migration assay, 2 × 10^4^ cells suspended in 100 μl serum-free DMEM were seeded in the upper compartment of the chamber and 800 μl DMEM with 10% FBS were added to the lower compartment of the chamber. The NC mimic or miR-107 mimic were added at the same time and the cells were incubated for another 24 h. After that, the cells were fixed with 4% paraformaldehyde for 30 min and stained with 0.1% crystal violet. The non-migrating cells in the upper chamber were removed carefully using a cotton swab. The migrated cells were cells on the lower surface were photographed with an Olympus IX70 inverted microscope in five randomly selected visual fields and the migrated cells were quantified using Image-Pro Plus 6.0 software. Each assay was performed at least three times.

For the invasion assay, the upper compartment was precoated with 100 μl of Matrigel. All other processes wer the same as for the transwell migration assay.

### Western blotting assay

The western blotting assay was conducted as previously described with some modifications [23]. Briefly, cells were treated with NC mimic or miR-107 mimic for 24 h, and then collected and lysed with RIPA buffer. After that, equal amounts of protein (50 μg) were subjected to western blotting assay. The bands were detected with an ECL Reagent Kit (Thermo Fisher Systems). β-actin immunoblots served as loading controls.

### Gene silencing and transfection with the miRNA mimic and vector

The cells were seeded in 6-well plates and cultured for 24 h. Then the cells were transfected with NC mimic and miR-107 mimic with Lipofectamine RNAiMAX transfection reagent (Invitrogen) according to the manufacturer’s protocol.

For the transfection of vectors, the cells were seeded in 6-well plates with a cell density of 1 × 10^5^ cells per well. After 24-h incubation, the cells were transfected with NC vector or TFR1 vector with Lipofectamine 3000 transfection reagent (Invitrogen) for 6 h. The cells were then cultured with normal growth medium for another 24 h and used for other experiments.

### Dual-luciferase reporter gene assay

The target genes of miR-107 were analyzed using the biological prediction site microRNA.org to verify whether TFR1 was the direct target gene of miR-107. Full-length cloning and amplification of the 3′-UTR region of the TFR1 genes was performed. The PCR products were cloned into multiple cloning sites of pGL4.49 vectors, which express firefly luciferase when activated, to form pGL4.49-TFR1-wt vector or pGL4.49-TFR1-mut vector. The 3′-UTR region of the TFR1 genes containing wild-type (WT) or mutant (MUT) binding sites of miR-107 were cloned into pGL4.49 [luc2p/TCF-LEF RE/Hygro] vectors. pGL4.73 [hRluc/SV40] vectors, which express Renilla luciferase, were used to control for transfection efficiency. The adherent cells were co-transfected with pGL4.49-TFR1-wt vector or pGL4.49-TFR1-mut vector and pGL4.73 [hRluc/SV40] vectors with Lipofectamine LTX & PLUS reagent following the manufacturer’s instructions.

After 24-h incubation, the cells were collected and luciferase signals were detected using a TECAN Infinite F500 platform with the Dual-Luciferase Reporter Assay System. Firefly luciferase activity was normalized to Renilla luciferase activity. The relative activity of the two luciferases was calculated (ΔCt). The experiment was performed three times.

### Statistical analysis

Data in each experimental group are presented as the means ± standard error of the mean (SEM) after analysis using GraphPad Prism 5.0 (GraphPad Software, Inc.). Significant difference between two groups was evaluated with two-tailed unpaired Student’s t-test, and significant difference between more than two groups was evaluated with one-way ANOVA followed by Tukey’s post hoc test. Statistical difference was considered significant when *p* < 0.05.

## Results

### MiR-107 is downregulated in colorectal cancer tissues and cells lines

**Fig. 1:**
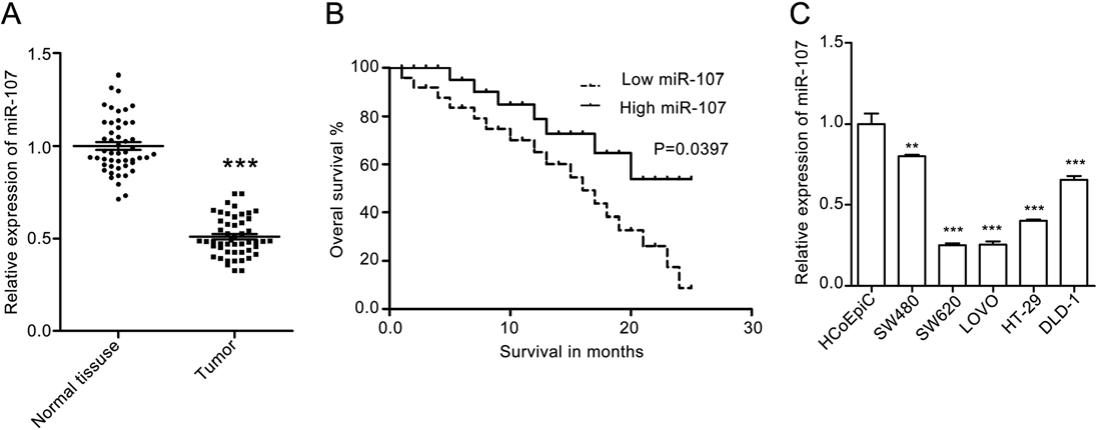
MiR-107 is upregulated in human colorectal cancer tissues. **a** The results of an RT-PCR assay for miR-107 expression in human colorectal cancer tissues and the corresponding normal mucosa tissues. The quantitative data are presented as the means ± SEM, *n* = 50. *** *p* < 0.001 compared with normal tissues. **b** Kaplan-Meier curves for overall survival for human colorectal cancer related to miR-107 expression. **c** The miR-107 expression in the normal human colon epithelial cell HCoEpiC and several human colorectal cancer cell lines. GAPDH was used for normalization of the miR-107 levels. Quantitative data are presented as the means ± SEM, *n* = 3. *** *p* < 0.001 compared with the HCoEpiC

First, we determined miR-107 expression in the colorectal cancer tissues and corresponding normal mucosa tissues using real-time PCR. We found that miR-107 is downregulated in colorectal cancer tissues compared with the level in normal mucosa tissues (Fig. 1a). Furthermore, the overall survival rate analysis (Kaplan-Meier method) showed that miR-107 expression was inversely correlated with patient survival rate. Patients with low miR-107 expression have a poor survival rate compared with patient with high miR-107 expression (Fig. 1b & Additional file 1: Table S1). We also determined miR-107 expression in the human colon epithelial cell line HCoEpiC and human colorectal cancer cell lines (LOVE, SW620, SW480, HT29 and DLD-1). The results showed that miR-107 expression in cancer cell lines is lower than that in the normal human colon epithelial cell line. Furthermore, miR-107 expression in SW620 and LOVE cells is lower than in the other colorectal cancer cell lines. We also found that miR-107 expression in SW480 is three timesfold of that in SW620 cells, indicating that the miR-107 level may correlate with the tumor metastasis (Fig. 1c).

### MiR-107 suppresses the proliferation and motility of SW620 cell

We next explored the role of miR-107 in the metastasis of colorectal cancers. Considering that miR-107 expression is lower in SW620 cells than in other colorectal cancer cell lines and that the SW480 and SW620 cell lines respectively represent primary colon tumor origin and lymph node metastasis, we selected SW620 for further in vitro research.

**Fig. 2:**
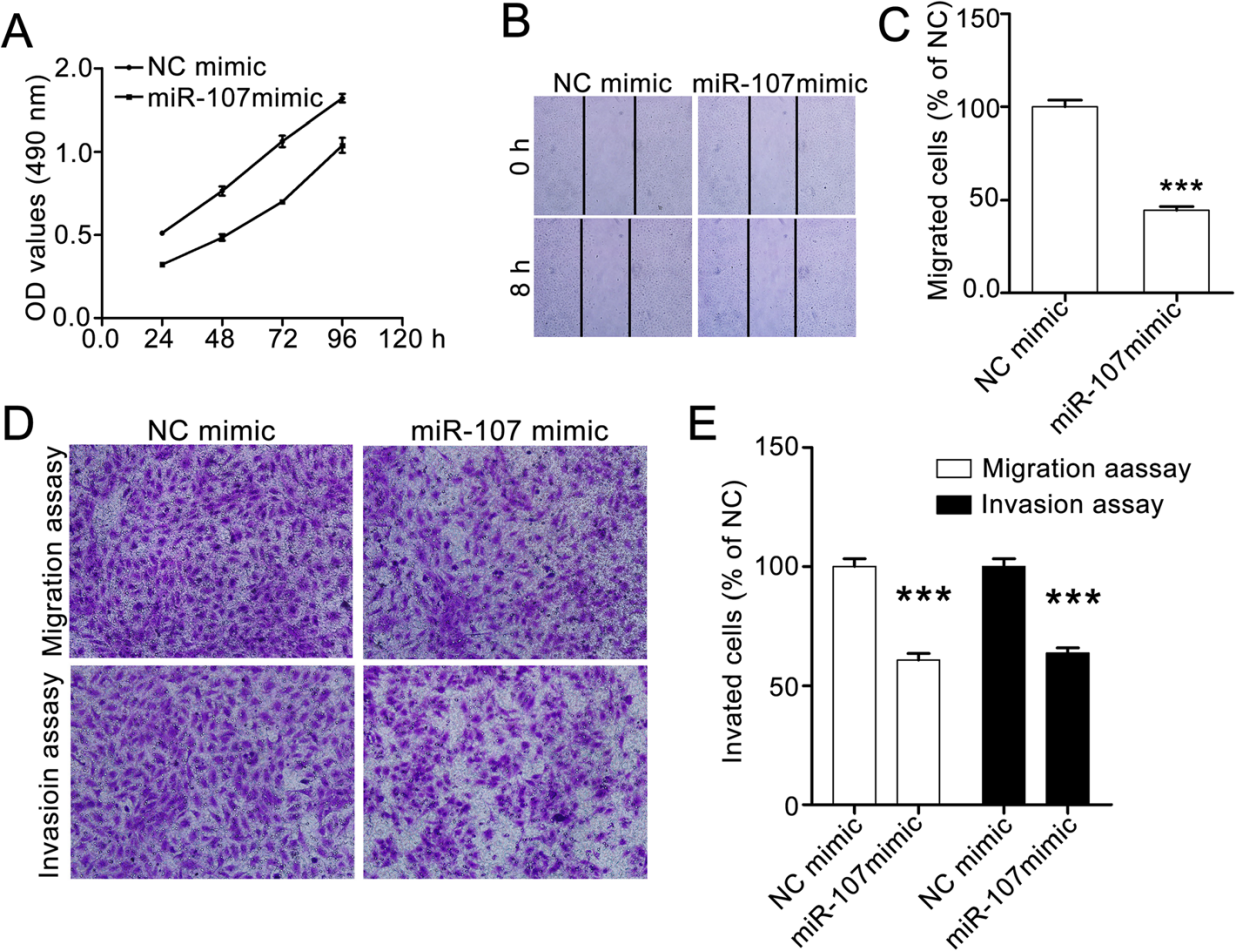
MiR-107 represses the proliferation, migration and invasion of SW620 cells. **a** MiR-107 suppresses the proliferation of SW620 cells. The cells were treated with NC mimic or miR-107 mimic for the times shown. Cell viabilities were detected using the MTT assay. **b** and **c** MiR-107 inhibits the horizontal migration of SW620 cells. The effect of miR-107 cells on horizontal migration was evaluated with a wound-healing assay. The confluent cells were starved with serum-free medium for 6 h and then scratched with 10-μl pipette tips. After washing with PBS, the cells were treated with or without miR-107 mimic for another 8 h. The images were taken at 0 h and 8 h in the same field with an Olympus IX70 inverted microscope. Representative images (100× magnification) and quantitative data are shown in (b and c), respectively. **d** and **e** MiR-107 supresses the vertical migration and invasion abilities of SW620 cells. The effect of miR-107 on vertical migration and invasion was assessed with transwell migration assay and tranwell invasion assay, respectively. Representative images (100× magnification) are shown in D and the quantitative data are shown in E. The data were analyzed with GraphPad Prism 5.0, and are presented as means ± SEM, n = 3. *** *p* < 0.001 compared with the control group

To evaluate the effect of miR-107 on SW620 cells, we treated the cells with NC mimic or miR-107 mimic for 24, 48, 72 and 96 h, then determined cell viability with the MTT assay. The OD value for the miR-107 mimic group is significantly lower than that in the NC mimic group, indicating that miR-107 repressed SW620 proliferation (Fig. 2a).

We then performed the wound-healing assay to estimate the effect of miR-107 on SW620 cell migration. We found that there were fewer migratory cells in the miR-107 mimic group than that in the NC mimic group, suggesting that miR-107 suppressed the horizontal mobility of SW620 cells (Fig. 2b and c). Similar results were observed in the transwell migration and invasion assays.

MiR-107 obviously inhibited the vertical migration and invasion of SW620 cells (Fig. 2d and e). Thus, our study suggests that miR-206 inhibits the proliferation and motility of A549 cells in vitro*.*

### MiR-107 directly target at TFR1

**Fig. 3:**
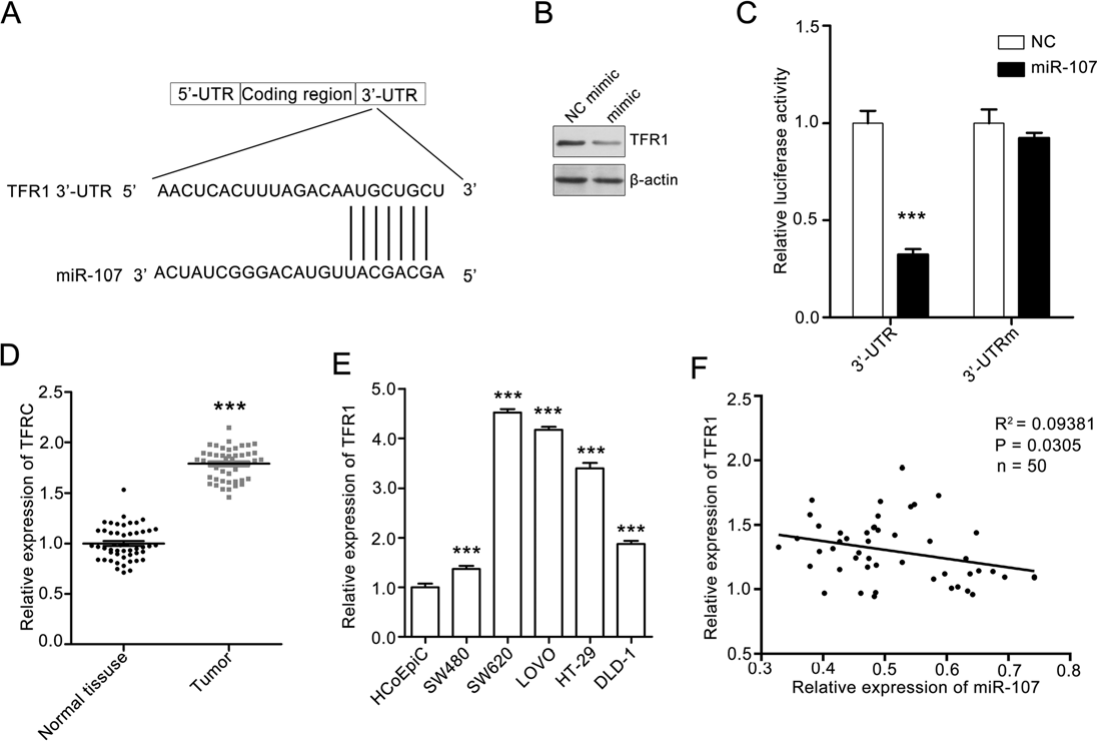
MiR-107 directly targeting at TFR1. **a** The sequence of human miR-107 and the predicted binding sites with miR-107 within the TFR1 3′-UTR are shown. **b** Photos of western blots showing that miR-107 inhibited the expression of miR-107 in SW620 cells. SW620 cell were treated with NC mimic or miR-107 mimic for 24 h. Then, the cells were collected and subjected to western blotting assay. **c** MiR-107 suppressed the transcription of TFR1 mRNA. SW620 cells were co-transfected with luciferase plasmids containing wild-type TFR1 3′-UTR or mutational TFR1 3′-UTR. The cells were also treated with NC mimic or miR-107 mimic. The cells were collected and lysed to measure the relative luciferase activity. Quantitative data are presented as the means ± SEM. *** *p* < 0.001 compared with the NC mimic group. **d** The TFR1 mRNA level in human colorectal cancer and the corresponding mucosa tissues. Quantitative data are presented as the means ± SEM, *n* = 50. *** *p* < 0.001 compared with normal tissues. **e** The TFR1 expressions in human colorectal cancer lines and normal human colon epithelial cell HCoEpiC were analyzed with RT-PCR. The TFR1 expression in HCoEpiC was set as 100%. Quantitative data are presented as the means ± SEM. *** *p* < 0.001 compared with BEA-2B cells. **f** The miR-107 expression in human colorectal cancer tissues is negatively correlated with that of TFR1. R^2^ stands for goodness of fit and p stands for significance of the slope

The above result showed that miR-107 may suppress colon metastasis. To further explore the underlying mechanism of miR-107-mediated SW620 cell behaviors, we predicted the mRNA targets of miR-107 using the TargetScan tool. There are more than 200 mRNAs that may be regulated by miR-107, including TFR1, which plays a vital role in tumor metastasis (Fig. 3a) [24]. Thus, we selected TFR1 for further studies.

We treated SW620 cells with NC mimic or miR-107, and then lysed the cells for a western blotting assay. As shown in Fig. 3b, miR-107 mimic treatment suppressed TFR1 expression. To further validate that miR-107 targets TFR1, we transfected SW620 cells with vectors containing the full-length wild-type or mutant 3′-UTR of TFR1, and conducted a dual-luciferase reporter gene assay. MiR-107 significantly decreased the luciferase activities of pGL4.49-TFR1-wt but had minimal effect on the luciferase activities of pGL4.49-TFR1-mut (Fig. 3c). All these results demonstrated that miR-107 directly targets TFR1.

Next, we performed RT-PCR to evaluate the TFR1 expression in human colorectal cancer tissues. We found that TFR1 was upregulated in colon tissues (Fig. 3d). Similar results were found in the RT-PCR assay of human colorectal cancer cell lines. The TFR1 expression is significantly increased compared with that in HCoEpiC cells and is higher in SW620 than in the other tested colorectal cancer cell lines (Fig. 3e). The correlation analysis showed that miR-107 expression was significantly inversely correlated with TFR1 expression in colorectal cancer tissues. The patients with low miR-107 expression tended to express higher levels of TFR1 (Fig. 3f). All in all, miR-107 acts as a tumor suppressor in human colorectal cancer by directly targeting TFR1.

### TFR1 overexpression attenuates the miR-107-mediated inhibitory effect on SW620 cells

To further estimate the role of TFR1 in the miR-107-induced suppressive effect on SW620 cells, we transfected the cells with TFR1 vector and then evaluated the effect of miR-107 on them.

**Fig. 4:**
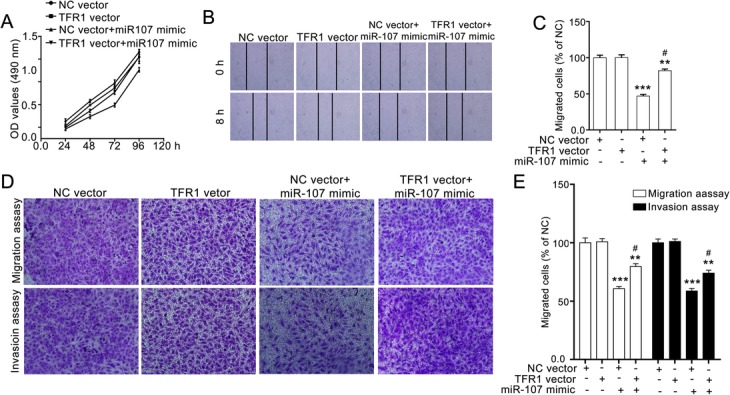
TFR1 overexpression restored the miR-107-mediated inhibitory effect on SW620 cells. **a** TFR1 overexpression attenuated miR-107-induced suppressive effect on SW620 cells. SW620 cells were treated with NC mimic or miR-107 mimics for 24 h, and then the cell viabilities were determined with the MTT assay. **b** and **c** TFR1 overexpression restores the miR-107-mediated effect on the horizontal migration of SW620 cells. SW620 cells were transfected with NC vector or TFR1 vector, and then used for the wound-healing assay. Representative images (100× magnification) and quantitative data are shown in (**b** and **c**), respectively. **d** and **e** TFR1 overexpression attenuates the miR-107-mediated suppressive effect on the vertical migration and invasion of SW620 cells. The SW620 cells were transfected with NC vector or TFR1 vector, and then subjected to transwell migration and transwell invasion assays. Representative images (100 × magnification) and quantitative data are shown in (**d** and **e**), representatively. Quantitative data are presented as the means ± SEM. *** *p* < 0.01 and *** *p* < 0.001 compared with NC vector group, ^#^*p* < 0.05 compared with NC vector+miR-107 mimic group

In the cell proliferation assay, we transfected SW620 cells with NC vector or TFR1 vector, and then treated the cells with miR-107 mimic. TFR1 overexpression obviously restored the miR-107-induced inhibitory effect on SW620 cells (Fig. 4a). We also found that TFR1 overexpression attenuated the miR-107-mediated inhibitory effect on the horizontal migration ability of SW620 cells (Fig. 4b and c).

Similar results were observed in the transwell migration and transwell invasion assays. TFR1 overexpression dramatically attenuated the miR-107-mediated suppressive effect on the invasion of SW620 cells (Fig. 4d and e). Thus, miR-107 inhibits the proliferation and motility of SW620 cells via targeting TFR1.

## Discussion

Although many advances have been made in the development of novel therapeutics for colorectal cancer, it remains one of the leading causes for tumor-related death worldwide, with 700,000 annual mortalities worldwide [25, 26]. Identifying key molecules contributing to colorectal cancer progress is essential for the future development of new and effective anti-colon cancer approaches [27]. In this study, we showed that miR-107 was vital for colorectal cancer progress, and explored underlying mechanism of miR-107-mediated inhibitory effect on the proliferation, migration and invasion of SW620 cells. Our study provides strong evidence for understanding mechanisms of colon cancer and contributes to the development of new anti-colon cancer progress.

MiRNAs are critical for the development and progression of colorectal cancer [28]. For example, miR-21, miR-26, miR-31, miR-141, miR-145, miR-196 and miR-200 were shown to be associated with the migration and invasiveness of colorectal cancer. Furthermore, the miR-200 family contributes to colorectal cancer stem cell-like properties [28, 29]. This stands as a reminder that identifying colorectal cancer-associated miRNAs and their target genes is critical for understanding the roles of miRNAs in colorectal cancer progression. Furthermore, miRNAs may be important from the point of view of novel therapeutic targets. Previous studies demonstrated that miR-107 was important for the progression of many tumors, including breast cancer, gastric cancer and pancreatic ductal adenocarcinoma [30–33]. However, whether miR-107 is involved in the process of colorectal cancer is still unclear. Here, we found that miR-107 expression is lower in colorectal cancer tissues than in normal tissues, and that low miR-107 expression often indicates a poor survival. Further research showed that miR-107 repressed the proliferation, migration and invasion of SW620 cells by targeting TFR1. Our study reveals the key role of miR-107 in colorectal cancer. In this regard, our study contributes to the identifying colorectal cancer-associated miRNAs and indicates that miR-107 is a potential target for colorectal cancer therapy. And we will evaluate the effect of miR-107 on SW620 xenografts and explore the underlying mechanism in the future.

TFR1, a transmembrane glycoprotein, is indispensable for iron import from transferrin into cells via endocytosis. Its expression is increased in many malignant tumors, such as breast, lung and bladder cancer and malignant gliomas [34, 35]. TFR1 has been considered as an attractive target for tumor therapy [36]. In this study, we found that TFR1 overexpression restored the miR-107-mediated inhibitory effect on SW620 cells. Our results also showed that TFR1 played critical role in the proliferation, migration and invasion of colorectal cancer cells. This indicates that TFR1 is important for the development of colorectal cancer. However, how miR-107 regulates TFR1 expression and the underlying mechanism of TFR1-mediated effect of SW620 cells are unclear, and we will explore the underlying mechanism in the future study.

In conclusion, miR-107 is downregulated in colorectal cancer and low miR-107 expression indicates a poor survival rate. MiR-107 inhibits the proliferation, migration, invasion of SW620 cells by targeting TFR1. Our study provides strong evidence that miR-107 is involved in the progression of colorectal cancer, indicating that miR-107 may be a promising molecular target in the therapy of colorectal cancer.

## Additional file

Additional file 1: **Table S1.** Correlation between miR107 levels and clinicopathological parameters for patients with colorectal cancer (DOCX 15 kb)

## Abbreviations

3′-UTR: 3′-Untranslated region
ATCC: American Type Culture Collection
DMEM: Dulbecco’s modified Eagle medium
FBS: Fetal bovine serum
GAPDH: Glyceraldehyde 3-phosphate dehydrogenase.
MDR: Multidrug resistance
miRNA: microRNA
MRP: Multidrug resistance-associated protein
MTT: 3-(4, 5-dimethylthiazol-2-yl)-2, 5-diphenyltetrazolium bromide
MUT: Mutant
PS: Penicillin–streptomycin
TFR1: Transferrin receptor 1
WT: Wild type
