# Supplementary material for: MiR-107 function as a tumor suppressor gene in colorectal cancer by targeting transferrin receptor 1
Source: Cell Mol Biol Lett. 2019 May 16;24:31. doi: 10.1186/s11658-019-0155-z (PMC6524234; doi:10.1186/s11658-019-0155-z)
Supplement: Supplementary file 1 — Table S1. Correlation between miR107 levels and clinicopathological parameters for patients with colorectal cancer (DOCX 15 kb) [file 11658_2019_155_MOESM1_ESM.docx]

Additional file 1: **Table S1.** Correlation between miR107 levels and clinicopathological parameters for patients with colorectal cancer

| Parameters | MiR-107 expression | | p value |
| --- | --- | --- | --- |
|  | High (n = 27) | Low (n = 23) |  |
| **Age** |  |  | 0.928 |
| ≤ 60 | 15 | 13 |  |
| > 60 | 12 | 10 |  |
| **Gender** |  |  | 0.653 |
| Men | 14 | 21 |  |
| Women | 9 | 6 |  |
| **Histological grade** |  |  | 0.009 |
| Well | 22 | 5 |  |
| Moderate or poor | 6 | 17 |  |
| **Clinical stage** |  |  | 0.028 |
| Ⅰ, Ⅱ | 23 | 6 |  |
| Ⅲ, Ⅳ | 4 | 17 |  |
| **Metastasis** |  |  | 0.001 |
| Yes | 4 | 19 |  |
| No | 23 | 4 |  |

The p value was calculated with a two-sided Pearson chi-square test.
